# Learned Experience and Resource Dilution: Conceptualizing Sibling Influences on Parents’ Feeding Practices

**DOI:** 10.3390/ijerph18115739

**Published:** 2021-05-27

**Authors:** Cara F. Ruggiero, Susan M. McHale, Ian M. Paul, Jennifer S. Savage

**Affiliations:** 1 The Center for Childhood Obesity Research, Penn State University, University Park, State College, PA 16802, USA; jfs195@psu.edu; 2 Department of Nutritional Sciences, Penn State University, University Park, State College, PA 16802, USA; 3 Department of Human Development and Family Studies, Penn State University, University Park, State College, PA 16802, USA; x2u@psu.edu; 4 Pediatrics and Public Health Sciences, Penn State College of Medicine, Hershey, PA 17033, USA; ipaul@pennstatehealth.psu.edu

**Keywords:** responsive feeding, siblings, family systems, parenting

## Abstract

Studies from diverse cultures report mixed results in the relationship between birth order and risk for obesity. Explanations may thus lie in the postnatal period when growth is shaped by the family environment, including parental feeding practices, which may be affected by siblings. Consistent with a family systems perspective, we describe two processes that may explain birth order effects on parental feeding practices and child outcomes: learned experience and resource dilution. Parents learn from experience when earlier-born children influence their parents’ knowledge, expectations, and behavior toward later-born siblings through their behaviors and characteristics—which can have both positive and negative implications. Resource dilution is a process whereby the birth of each child limits the time, attention and other resources parents have to devote to any one of their children. The goal of this review is to provide a theoretical basis for examining potential sibling influences on parental responsive feeding toward developing recommendations for future research and practice aimed at preventing obesity throughout family systems.

## 1. Introduction

A body of research provides evidence of associations between birth order and health, achievement, and behavior [1,2,3]—an important pattern given that approximately 80% of U.S. children <18 years old have at least one sibling [4]. Relevant to weight-related outcomes, studies from diverse cultures report that firstborns have a higher prevalence of obesity than later-born siblings. This finding is somewhat paradoxical given that later-born siblings are more likely to experience an adverse prenatal intrauterine environment characterized by higher maternal pre-pregnancy BMI and/or gestational diabetes—both associated with higher birthweight [5,6,7,8,9,10]. In contrast, other studies report that the youngest children are more likely to be overweight or obese [11,12,13,14], suggesting the need to understand mechanisms that explain birth order effects. In addition to birth order, “sibling influences” are evident in findings showing that sibship size (i.e., the number of siblings in a family) is inversely associated with overweight and obesity [9], and further, that children without siblings (only children) are more likely to be overweight/obese than firstborns with one or more sibling(s) [9,11,15]. These effects of sibship size may be due to differences in siblings’ health behaviors given findings of positive associations between sibship size and physical activity and both healthy sleep and dietary habits [15]. Taken together, such results suggest that siblings may play a key, albeit indirect, role in childhood overweight and obesity. The focus on structural characteristics (i.e., birth order, sibship size), however, means that researchers and practitioners are left to speculate about the potentially malleable family processes through which these structural factors have their impacts. In this paper, as a step toward illuminating sibling-related family dynamics that may underlie birth order differences in childhood overweight, we focused on two sibling-related family systems processes—learned experience and resource dilution—and their potential role in sibling differences in parental feeding practices.

### 1.1. Learned Experience

A family systems perspective [16], which highlights reciprocal influences between and among family subsystems, offers a roadmap for research and practice. For example, what parents learn from their experiences with an earlier-born child may influence their behaviors toward a later-born child [16]—a sibling-related family systems process termed learning from experience [17]. Through this process, the behaviors and characteristics of an older sibling may influence parents’ knowledge and expectations for later-born siblings—in both positive and negative ways. In one prior study, for example, parents who experienced a child’s “easy” transition to adolescence were less likely to expect transition difficulties in the younger sibling [18], suggesting that experiences with an older child, not simply that child’s presence, explained parents’ expectations for the younger sibling. Other studies of parents’ learned experience have documented more effective parenting of mothers and fathers toward a secondborn as compared with firstborn adolescent siblings in the domains of parent–child conflict, warmth, and knowledge of their child’s whereabouts, companions and everyday experiences [17,19,20]. Relevant to learned experience, Reiner, Hess and colleagues found that mothers who reported high self-efficacy but low knowledge were the least sensitive with their infants and suggested that these mothers were naively confident about their parenting abilities—findings that underscore the importance of knowledge that can be gained from the experience with a firstborn [21]. To our knowledge, however, the learned experience process has not been studied in reference to parental feeding practices.

### 1.2. Resource Dilution

Another sibling-related family systems process is resource dilution, whereby the birth of each subsequent child means that parents must divide their time and resources among more children [22,23]. Resource dilution effects may be especially apparent with shorter spacing between siblings’ births [24,25]. In turn, research based on multiple national datasets in the U.S. [25,26,27], Europe [28], and Asia [29] suggests that children later in birth order exhibit lower educational attainment. Sibship size also is negatively associated with some outcomes of child well-being in earlier-born children [30,31]. For example, studies of only children are in accord in showing that only children perform better academically and that their social outcomes are similar to those with siblings [30,31]. Consistent with a resource dilution explanation of birth order effects on achievement, one study showed that parents exhibited less demanding academic expectations for later-born children [32], and another showed that mothers of secondborns were less warm and involved than mothers of firstborns [28]. As we elaborate below, a limitation of this work is its reliance on between-family designs (i.e., comparing the experiences and outcomes of earlier-born siblings in one group of families to those of later-born siblings in other families). In contrast, a within-family design enables researchers to test whether the same parent behaves differently toward an earlier-born child than she/he does toward a later-born child. By treating each parent as his/her own “control” in this way, family background differences or variables confounded with sibship size can be ruled out, allowing for stronger inferences about sibling differences.

### 1.3. Parental Responsive Feeding Practices

The quality of parenting in early life influences children’s health and development [33]. Responsive parenting, defined as responding to children promptly, contingently, and in ways that are developmentally appropriate [34,35,36], is associated with a range of positive child outcomes [37,38,39,40]. Drawing from the broader parenting literature, this paper will discuss parental feeding practices in the context of responsive feeding, a component of responsive parenting. Responsive feeding includes three components: (1) perception of the child’s cues (e.g., hunger; fullness), (2) accurate cue interpretation, and (3) appropriate cue responses [41,42]. For example, (1) an infant may cry when hungry, (2) the parent will interpret this cry as hunger after trying other soothing strategies, and (3) the parent will respond and provide a bottle or breastfeed. In turn, responsive feeding is thought to promote the development of appetite regulation (i.e., eating in response to hunger cues and not eating beyond fullness) and healthy growth [41,43,44,45,46], with appetite regulation hypothesized to play a role in the link between parental feeding practices and child weight status [45]. However, nonresponsive practices such as responding to infant cries using control-based practices such as food to soothe [47] may inhibit a child’s ability to learn to regulate their appetite and increase risk for outcomes such as overeating, weight gain, and obesity later in childhood [48,49,50,51]. Of note, some work shows that mothers tailor their practices based on the needs and characteristics of the individual child—including child traits and behaviors such as distractibility, negative mood [52], and eating behaviors [53]. Such findings suggest, in turn, that parents may treat siblings differently when it comes to feeding. Little to nothing is known about these sibling differences and influences on parental responsive feeding practices. 

Adopting a family systems perspective that considers learned experience and resource dilution, while expanding beyond a focus on more than one child per family [54], may serve to advance the literature on parental feeding practices and obesity risk. Almost nothing is known about sibling differences in parental responsive feeding [55,56] or the circumstances under which these differences emerge. Such an approach may help to illuminate the mechanisms underlying birth order effects on overweight and obesity as evidenced by systematic reviews [9,15] and, on a practical level, provide anticipatory guidance to parents that targets family systems dynamics. In sum, grounded in a family systems perspective, we focused on learned experience and resource dilution processes as potential explanations of sibling differences in parental responsive feeding in early life, and further, we examined the role of child characteristics in these processes.

## 2. Sibling Influences on Parental Responsive Feeding Practices to Illuminate Obesity Risk

### 2.1. Sibling Influences

Siblings are central in the lives of children. As building blocks of the family, siblings can influence one another indirectly through their effects on larger family dynamics by virtue of their family niche (e.g., the lastborn, the athlete) [57], or through ripple effects of their behaviors and experiences throughout the family system—such as when a picky eater affects family meal time dynamics [57,58]. Siblings also can influence one another directly as companions, role models or foils, and competitors [57]. In the early 1980s, Dunn and others argued for the importance of examining dynamics like these to explain the effects of sibling structure factors, including sibling gender constellation, age spacing, sibship size and birth order [59]. As these early sibling researchers explained, findings on the effects of structural factors accounted for limited variance in sibling outcomes, because these factors do not always set particular family processes into motion, and it is the family processes that have effects on children. It is important to note that family processes may be malleable and thus, unlike structural factors, potential foci for intervention. 

These kinds of sibling-related family dynamics can have effects on childhood overweight/obesity via parental feeding practices. For example, the learned experience process may help to explain birth order effects, such as when parents who may have overfed their earlier-borns due to (misguided) concerns about their weight status, come to understand that their vigilance is unnecessary and are more relaxed about their later-borns’ feeding. Parents may also learn from experiences with their earlier-borns, how to better read and respond to infant hunger and fullness cues. In such ways, their learning may have implications for parenting of later-born children and lead to better outcomes for later-borns with respect to overweight. A resource dilution process also may explain sibling differences in overweight and obesity, but suggests a different pattern of outcomes. From this perspective, children later in the birth order hierarchy may be exposed to more obesogenic parental feeding practices because their parents are more pressed for time (i.e., parent responsiveness in feeding may decrease with each successive child). On a practical level, integrating such family systems processes has implications for the design of interventions, specifically, incorporating a focus on these larger family dynamics as targets for evaluation and potential change. A key research direction, however, is to identify the circumstances under which these family processes emerge and have their effects. For example, in some families, learned experience leading to increased responsiveness in feeding may explain family dynamics best, because parents become more comfortable with their roles and thus less controlling of their children’s eating, which may lead to better appetite regulation and healthier weight status. Conversely, resource dilution may dominate in other families such as when parents experience financial stress or time constraints and children are exposed to less healthy food choices (e.g., more fast food, fewer fresh fruits and vegetables) or less family meal time routines, thus increasing risk for obesity. Families also may experience both of these processes simultaneously, learning from experience in some aspects of responsive feeding (e.g., the importance of routines), but dilution in others (e.g., increased use of food to manage behavior). 

### 2.2. Learned Experience and Parental Responsive Feeding

With respect to learned experience, although parental learning may imply benefits for later-born siblings, prior research reveals that this process is not universal, nor are the outcomes for later-borns always superior to those of earlier-born siblings. For example, a study of the transition to adolescence revealed that parents’ experiences with their older child had implications for their expectations regarding a younger sibling only when the siblings were similar in temperament [18]. In a related study, parents who had experience with an earlier-born adolescent’s transition were less likely to expect increases in conflict and other difficulties in their later-born—but they also were less likely to expect intimacy and closeness with their child [60]. Similarly, parents’ learned experience may have both positive and negative implications for their parenting and feeding of later-borns. For example, if their earlier-born child exhibits rapid weight gain, parents may learn that using food to soothe infant distress leads to weight gain, causing them to avoid this practice with later-born children. In contrast, an earlier-born with a large appetite may lead parents to use food to soothe or food as reward in response to child distress or to manage behavior given its effectiveness with a child who is very responsive to food cues. Parents may then use this control-based feeding strategy with a later-born child, regardless of the child’s own appetite. 

### 2.3. Resource Dilution and Parental Responsive Feeding

Similarly, although resource dilution implies that later-borns will be disadvantaged, the nature and extent of “dilution” and its implications may vary across contexts [61]. Additionally, most research on resource dilution focuses on parental resources [61] and conceptualizes siblings as risks. Siblings, however, can serve as positive role models (e.g., of healthy eating), gatekeepers (who introduce their siblings to sports and other physical activities) and caregivers. Further, resource dilution and its effects may not be monolithic, but may vary based on the type of resource being diluted—such as parental time, responsiveness, finances or other family resources. For example, a study of the effects of the birth of a sibling revealed that dilution of “interpersonal resources” (e.g., parent–child interactions, maternal mental health) was more strongly linked to young children’s cognitive development than dilution of material resources (e.g., financial) [62]. Another study showed that the birth of a sibling was linked to declines in mothers’ positive interactions with their children in support of the resource dilution hypothesis; in contrast, the extent of consistent parenting increased [63], suggesting that some parenting practices may be affected more than others in different family contexts and resources may be reallocated instead of simply diluted. For example, children’s meal and snack routines may remain intact after the birth of a sibling in family contexts with more social supports: Feeding routines can be established or enhanced through childcare settings, partner support, or the models provided by older siblings, all of which contribute to the family support system.

## 3. Roadmap: Reciprocal Family Systems Processes and Parental Responsive Feeding

**Figure 1:**
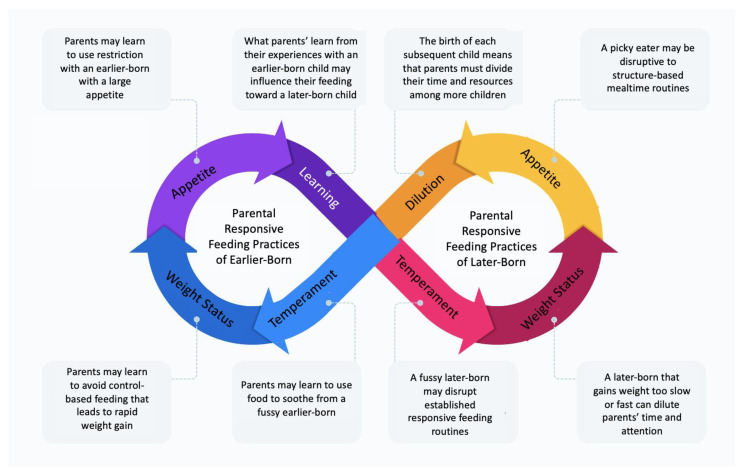
Conceptual model: The role of sibling-related family systems processes-learned experience and resource dilution—in parental responsive feeding.

Grounded in the family systems literature, a theoretically informed model of sibling influences on parental responsive feeding practices is shown in Figure 1.

The premise of this model is that sibling characteristics influence the parental responsive feeding of a child over and above that child’s own characteristics. That is, parents’ experiences gained from having an earlier-born child, as well as that child’s characteristics (i.e., weight, temperament, appetite), may influence how later-born children are fed through a learned experience process. Additionally, both the experience of having a later-born child and that child’s characteristics may affect how earlier-borns are fed through a resource dilution process or learned experience. This hypothesis is in contrast to existing resource dilution literature in that we are proposing that there can be risk for earlier-borns as well as family size increases. These processes are not mutually exclusive and may coexist through a reciprocal process. For example, the pickiness of a later-born child could dilute healthy food choices, causing parents to provide a lower variety of foods to all children in the household at mealtimes. Alternatively, that same picky later-born could also allow parents to learn how pressure can backfire at mealtimes, leading them to avoid using it with other children in the household. 

### 3.1. Evidence of Learned Experience

Our conceptual model builds on existing responsive feeding literature by incorporating parental responsive feeding practices with more than one child. As is also shown in our conceptual model, child characteristics, such as temperament, have been shown to be associated with parent feeding [51,64]. Our model extends what is understood about the role of child characteristics by incorporating the role of siblings and their characteristics in parental feeding practices. In this way, our model acknowledges the central role of siblings in family systems by incorporating sibling influences on parental responsive feeding. To our knowledge, research on parental feeding practices has not directly addressed these sibling-related family dynamics, but some studies have provided evidence of their operation. With respect to learned experience, classic studies from the 1970s suggested that first-time mothers were less responsive to hunger and fullness cues with firstborns, and multiparous mothers were more responsive with later-born infants [65,66,67]. Further, first-time mothers fed their older infants longer and more frequently than did multiparous mothers [68]. When applying a learned experience process to feeding, however, it will be important to consider the potential for learning maladaptive practices that can spill over to later-born children. For example, a fussy earlier-born may promote controlling feeding practices, such as using pressure or food to soothe, that are then used with later-born children. 

### 3.2. Evidence of Resource Dilution

Although there is a substantial literature on the transition to parenthood [69], surprisingly little is known about the arrival of a sibling—especially in in the realm of responsive feeding. No studies to date have examined this. However, few studies on sibling arrival and weight status may offer some insight. This existing literature is inconsistent with the hypotheses of a resource dilution model: Although the prediction might be that child BMI would increase when a sibling is born due to parents engaging in less-responsive feeding (consistent with findings that firstborns have more overweight and obesity, on average, than later-borns), some literature comparing only children and those with a sibling shows the opposite. Two studies that examined BMI trajectories in school-aged children found that the birth of a sibling [12,70] was associated with a lower BMI *z*-score trajectory in the firstborn child compared to those who did not have a sibling. These findings that having a secondborn sibling is associated with a healthier BMI *z*-score trajectory and that only children are at greater risk for obesity compared to firstborns with a sibling are consistent with other obesity literature [9,15], in that sibship size is inversely associated with BMI. The latter study tested but found no mediation by obesogenic-relevant behaviors such as screen time, active play time, family dinner frequency, or diet quality, suggesting that other factors may be at play [70]. For example, the demands of a larger family may, in fact, dilute parents’ resources but take the form of lessening their control over their children’s eating. A learned experience process also may be possible, such as when parents become more comfortable in their roles with the birth of a second child. An important research direction is to longitudinally test the characteristics of children and their siblings as potential influences on parental feeding practices with each—as shown in Figure 1.

### 3.3. The Role of Sibling Characteristics in Parental Responsive Feeding

As noted, our model extends prior research on the role of children’s own characteristics influencing parental responsive feeding to take into account the characteristics of their siblings. Siblings differ in many ways, including sex, age, interests, and temperament [71]. The latter domain, temperament, encompasses characteristics such as reactivity, self-regulation, sociability, and mood. Although individual differences in temperament are evident early in life, they are modifiable by the environment [72]. With respect to feeding in early life, children who are more reactive, fussy, or negative in mood are more likely to experience nonresponsive, control-based parental feeding practices such as the use of food to soothe distress, a feeding practice that, as noted, can negatively impact children’s weight [73,74]. For example, a study of school-aged children revealed that parents reported using more restriction in children with more negative moods [52]. If parents learn from experience, those with fussy earlier-borns may use these same obesogenic feeding practices with later-born children, because they have proven effective in the past. This learning may also be positive, however, as when the experience of having an earlier-born gives parents practice in recognizing and appropriately responding to their children’s hunger and fullness cues. Further, earlier-borns with “easy” temperaments may facilitate this learning, allowing parents to more readily learn to recognize hunger/fullness cues and thus develop responsive feeding practices that they then use with later-borns. Child temperament also may play a role in resource dilution processes, as when a fussy later-born places extra demands on parents and disrupts responsive feeding of other siblings: parents whose time and energy are stressed by a child with a “difficult” temperament may resort to more controlling feeding practices with other children or to easier and more convenient (e.g., fast food) meal choices. 

Child weight status is another sibling characteristic that may influence parents’ concerns about their children’s eating behaviors and, in turn, their feeding practices given that weight concerns are a strong predictor of parental feeding practices [75]. A study of twins and a second, qualitative study documented that mothers use different feeding practices with their children based on each child’s weight status [55,76]. Further, discordant sibling designs can be used to study variability in parental feeding practices. However, despite differing weight concerns, most parental feeding practices of normal weight and overweight siblings have been shown to be similar [77,78,79], suggesting that more dynamics within a family may be at play. Building upon this literature with a focus on a learned experience process, parents with earlier-borns who are at risk for overweight may continue to use more restrictive feeding with their later-born children, beyond what would be expected based on the later-borns’ own weight status. More longitudinal studies are needed to test this hypothesis.

Finally, children’s appetitive traits (e.g., food responsiveness, satiety responsiveness) are another set of characteristics that may influence parental feeding of siblings. Such characteristics have been linked to both parental feeding practices [53,64,80,81,82] and child weight [83]. Sibling appetite, in turn, may have implications for parent feeding. Although this literature demonstrates how parents respond to each child’s characteristics, these studies did not consider larger family dynamics and the influence of sibling characteristics on the feeding of the other child. Therefore, it could be hypothesized that the appetite of one child may influence how responsive a parent is in feeding other children in the household. For example, an earlier-born picky eater could cause parents to develop pressure-to-eat feeding practices that are then used with later-born children, regardless of their own pickiness. Alternatively, a later-born child who is very responsive to external food cues could promote more snacking for all children in the household. Taken together, these foundational findings on the role of child characteristics in parents’ feeding practices lay the groundwork for studies of their potential role in how parents feed their siblings—such as through the operation of learned experience and resource dilution processes. 

## 4. Implications for Family-Based Obesity Prevention Research

While there is existing literature on birth order, sibship size and obesity, cross-sectional studies examining parental feeding practices (most often of one child), and interventions examining responsive feeding as a strategy for obesity prevention, study designs have not yet explored these simultaneously. Findings reviewed above suggest the need to move beyond a focus on one child per family to design effective responsive feeding interventions for entire families and to acknowledge that child characteristics may influence the parenting practices that their siblings experience. Obesity and family researchers need to better understand the circumstances under which sibling differences in parental feeding practices emerge by focusing on sibling-focused messaging, family transitions, specific child characteristics as well as contextual influences. For these reasons, we have highlighted implications for future research and novel targets for family-focused intervention. These expand upon our conceptual model and build on prior research on parental feeding practices including the role of child characteristics. Implications are summarized below and shown in Table 1.

Although responsive feeding is best for all children, it is well established that child characteristics are associated with parental feeding practices [64,73,74]. As we have discussed, children’s appetites may develop as a result of parental feeding practices, and parents may also feed in response to a child’s individual characteristics [84]. We know almost nothing, however, about whether and how the characteristics of one child affect parents’ feeding practices with siblings. Following systematic study of sibling-related family systems processes such as learned experience and resource dilution, a next step will be to expand interventions to consider the characteristics of other children in the family and how they may affect the feeding of a target child. That is, intervention messaging for family-based interventions should come from the lens of a family systems perspective. Additionally, many responsive feeding interventions to date have focused on firstborns in infancy. Future interventions could consider booster messages for later-born children that consider children’s individual characteristics and the larger family context. 

At the transition to parenthood, parents must adapt their own behaviors and activities and reorganize their families to address new roles and responsibilities [85]. These transition periods may be marked by greater stress, conflict and risk for psychological problems [86]. Yet, times of transition may be opportunities for change, and therefore, these periods may be the most effective times to intervene [87]. Incorporating information on the larger systems within which families are embedded—child care and school, parents’ workplaces, health care, social programs, as well as formal and informal supports for families—can further illuminate resources and challenges that have implications for parental feeding practices across the course of family development.

As we have argued, a gap in the current parenting and feeding literatures is information on what parents learn from their earlier experiences that may—either negatively or positively—influence their parenting behaviors toward later-born children. Although learned experience has been studied to explain birth order, parents may also learn from their experiences with later-borns and apply those lessons to earlier-born children. For example, parents may feel more confident after parenting later-born children and for reasons of efficiency they may develop more regular meal and snack routines, which benefit the entire family. Key research directions include using within-family comparisons to determine whether parents differ in their feeding practices as a function of birth order and, more importantly, to identify factors that account for differences in parental feeding practices, including, for example, increased knowledge and comfort or reduced anxiety in the parenting role. Such information has implications for intervention targets. Turning to resource dilution, most prior literature has drawn inferences about the operation of this process based on birth order differences in child outcomes, but failed to directly measure what resources are limited, for whom, and under what conditions [62]. Thus, an important research direction is to assess whether and what resource dilution emerges in the feeding context, including mealtime routines, food exposure and parental feeding practices, as well as the factors, including sibling and family context characteristics that give rise to resource dilution—or, correspondingly, support parental responsive feeding practices and healthy eating throughout the family. In turn, educating parents about strategies to protect against dilution when a later-born child arrives can help parental responsive feeding practices to remain intact. Examples of these strategies may include giving earlier-born children jobs to help out at mealtimes, feeding all children and family members the same foods, adjusted to be developmentally appropriate (e.g., portion sizes), or reorganizing parenting roles (e.g., one partner cares for a newborn while the other partner cares for older siblings). Finally, although a focus on the broader context is beyond the scope of this review, intervening at multiple levels of influence can bolster efforts to achieve positive impacts on family systems processes [61,88].

## 5. Summary and Conclusions

This paper described two sibling-related family systems processes (learned experience and resource dilution) that may have implications for parental feeding practices and, in turn, for obesity prevention within families. By taking into account children’s and siblings’ characteristics and directly assessing the processes of resource dilution and learned experience, investigators may advance understanding of parental feeding practices. In turn, studies of the conditions under which learned experience and resource dilution emerge and operate can provide information for incorporation into existing parent education programs that target responsive feeding practices. More generally, to better understand and support parents, researchers and practitioners should move beyond a focus on one child per family, as is common in much of the obesity and responsive feeding literatures. Within-family designs and longitudinal approaches should be adopted to explore how the experiences and characteristics of multiple family members and subsystems are mutually influential. From a family systems perspective, such an approach is key to understanding, not just how families operate, but the health and well-being of individual family members as well [89].

## Figures and Tables

**Table 1:** Directions for future research and practice to address sibling influences on parental feeding practices.

Target	Future Directions
**Focus on Siblings**	
Responsive feeding is best for all children; however, children have unique characteristics and what worked for one child may not work for another.	Provide booster messages in responsive feeding interventions that highlight factors that should be considered in parenting later-borns due to their different characteristics. For example, a fussy later-born may require additional soothing strategies to avoid using food to soothe—strategies that parents did not need to use with a calmer firstborn.
The arrival and characteristics of later-borns can affect established routines with earlier-borns.	Incorporate strategies aimed at reducing dilution with the arrival of later-born children, such as giving jobs to earlier-born children and re-organizing the inter-parental division of labor. Tailor strategies based on later-born children’s characteristics (e.g., temperament, appetite, etc.).
**Examine Longitudinal Changes in Feeding Across the Transition to Siblinghood**	
The parenting literature has focused on the transition to first-time parenthood, but less is known about the arrival of later-born siblings, especially as it relates to feeding.	Conduct studies of parental feeding practices before and after the births of siblings to identify strains related to feeding routines for future intervention targets.
**Determine What Parental Feeding Practices and Child Characteristics are Most Prone to Learning and Dilution**	
It is unknown what feeding practices are most susceptible to learning and to dilution.	Determine what parental feeding practices are most susceptible to learning from experience and resource dilution to guide intervention development.
It is unknown which child characteristics may have implications for learning and dilution.	Determine which sibling characteristics may contribute to positive and negative learning and are most disruptive or promotive of parental responsive feeding.
**Incorporate Larger Contextual Influences and Supports**	
Social programs may support positive learning and reduce resource dilution.	Examine associations between food safety net programs, learning from experience and resource dilution in feeding. Targeted messages may be provided through these programs, and receipt of nutrition safety net benefits can be explored as a moderator of intervention effects on learning or dilution.
Intervene at multiple levels for stronger impacts.	Incorporate workplaces, places of worship, and childcare/school facilities into interventions to increase support for families to protect against dilution and encourage positive learning.

## Data Availability

Not applicable.
